# Roots: metabolic architects of beneficial microbiome assembly

**DOI:** 10.1093/plphys/kiaf349

**Published:** 2025-08-05

**Authors:** Melissa Uribe-Acosta, Alberto Pascale, Jiayu Zhou, Ioannis A Stringlis, Corné M J Pieterse

**Affiliations:** Plant-Microbe Interactions, Department of Biology, Science4Life, Utrecht University, Utrecht 3508 TB, the Netherlands; Plant-Microbe Interactions, Department of Biology, Science4Life, Utrecht University, Utrecht 3508 TB, the Netherlands; Institute of Botany, Jiangsu Province and Chinese Academy of Sciences, Nanjing 210014, China; Plant-Microbe Interactions, Department of Biology, Science4Life, Utrecht University, Utrecht 3508 TB, the Netherlands; Laboratory of Plant Pathology, Agricultural University of Athens, Athens 11855, Greece; Plant-Microbe Interactions, Department of Biology, Science4Life, Utrecht University, Utrecht 3508 TB, the Netherlands

## Abstract

The increasing demand for sustainable agricultural practices has driven a renewed interest in plant–microbiome interactions as a basis for the next “green revolution.” Central to these interactions are root-derived metabolites that act as mediators of microbial recruitment and function. Plants exude a chemically diverse array of compounds that influence the assembly, composition, and stability of the root microbiome. These metabolites can act as nutrients, chemical signals, or antimicrobial barriers, orchestrating beneficial relationships while defending against pathogenic threats. This review highlights the multifaceted role of plant metabolites in root microbiome assembly, focusing on their dynamic regulation by plant genotype, environmental conditions, and immune responses. We discuss the emerging concept of roots as metabolic architects of their associated microbiomes, wherein plant–metabolite–microbiome interactions coevolved alongside critical life-support systems such as immunity and nutrient acquisition. We propose that elucidating the mechanisms of metabolite-driven microbial selection can guide the development of future crops optimized for beneficial microbiome recruitment and enhanced resilience.

## From green revolution to the microbiome era

The green revolution of the 1960s, driven by synthetic fertilizers, pesticides, and improved crop varieties, increased the agricultural productivity of key crops by 40% to 50%. However, its environmental toll has underscored the need for more sustainable strategies (Renaud et al. 2018). One promising alternative lies in harnessing the symbiotic relationship between plants and soil-inhabiting microbes (Bakker et al. 2020; Banerjee and van der Heijden 2023). These microbes and their functional capacities are collectively referred to as the soil microbiome (Berg et al. 2020). They play a crucial role in supporting plant nutrition and enhancing resistance to both biotic and abiotic stresses (Berendsen et al. 2012, 2018). Therefore, optimizing microbiome-based strategies has been proposed as the foundation for a potential “second green revolution,” one that aims to reconcile the dual goals of food security and environmental sustainability.

## The root–soil microbiome interface: gatekeeper of plant health

The soil microbiome comprises a vast array of organisms, including beneficial, commensal, and pathogenic microbes (Poppeliers et al. 2023). Its composition influences plant health and productivity (Carrion et al. 2019; Spooren et al. 2024). In fact, using artificial intelligence approaches, variations in microbiome composition across agricultural fields have been shown to reliably predict plant health (Wei et al. 2019; Lutz et al. 2023; Song et al. 2025). Soil microbiomes provide over 40 functions relevant to ecosystem health (Banerjee and van der Heijden 2023). The microbiome thus acts as an extension of the plant's own genetic repertoire, often referred to as the “second genome” (Henry et al. 2021) and is increasingly viewed as part of the holobiont: a host organism and its associated microbial community, which together may function as a unit of evolutionary selection (Theis et al. 2016).

Although plants can derive numerous benefits from microbial communities, they must also exert strict control over these associations to maintain microbial balance and prevent harmful disruptions (Chen et al. 2020; Nakagami et al. 2024). The plant's influence on the composition of the soil microbiome is referred to as the plant host-genotype effect (Box 1). This plant-driven selection process reduces microbial diversity as soil microbial communities approach the roots. Three sequential, spatially defined compartments are recognized as crucial to this selection process: the rhizosphere, the rhizoplane, and the endosphere (Box 1) (Reinhold-Hurek et al. 2015). The rhizosphere is rich with root exudates, including a variety of metabolites that influence the soil microbiome by acting as chemoattractants, carbon or nutrient sources, antimicrobials, or chemical defense compounds (Chagas et al. 2018; Knudsen et al. 2018; Sasse et al. 2018; Pascale et al. 2020; Pang et al. 2021; Hong et al. 2022; Yu et al. 2022).

Box 1. Root zones and the host genotype effect. Plant roots exert a host genotype-dependent selective pressure on soil microbial communities. The illustration provides a magnified view of the soil region influenced by plant roots, the rhizosphere. The plant root (left) features chemical and structural barriers that regulate interactions with the surrounding microbiome. Structural and chemical barriers (mainly secondary metabolites) can prevent microbes from entering the root tissues; therefore, only a small subset of soil microbes can establish on the root surface (the rhizoplane), and even fewer can enter the interior root tissues (the endosphere). Microbes positively selected by the plant root are depicted as blue cells in the figure. The root also modifies the rhizosphere microbiome (center) through the exudation of diverse metabolites, including primary metabolites that provide nutrients to the microbial community and secondary metabolites with selective antimicrobial properties, acting as chemical barriers. As a result, the microbiome composition in the rhizosphere differs from that of the bulk soil (right), depicted here as a higher proportion of blue microbes in the rhizosphere and greater presence of red microbes in the bulk soil. Created in BioRender: https://BioRender.com/1hmyyss.

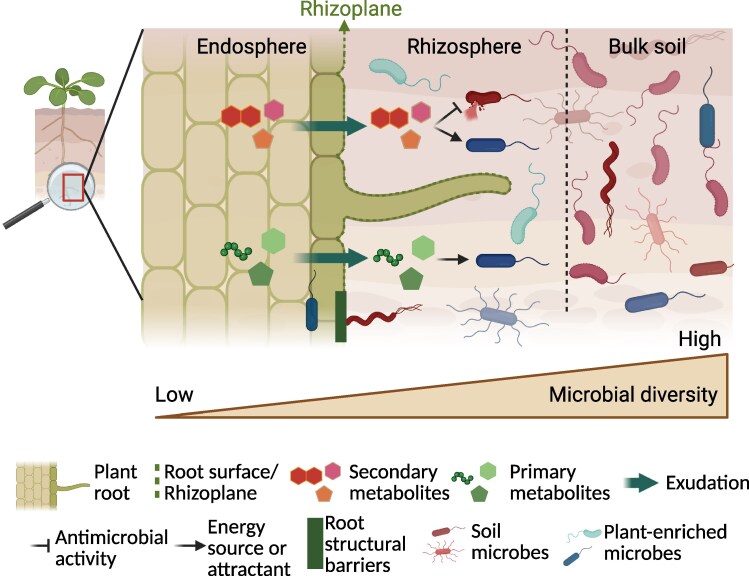

At the rhizoplane, microbes adhere to the root surface, encountering root exudates and plant immune responses (Reinhold-Hurek et al. 2015). The endosphere is a highly selective environment that permits colonization by only a limited number of microbial taxa (Edwards et al. 2015). Structural barriers such as lignin and suberin, present in the rhizoplane and endosphere, can physically hinder microbial colonization of specific root zones (Froschel et al. 2021). The influence of the host genotype diminishes with increasing distance from the roots (Bulgarelli et al. 2013). Consequently, environmental factors exert a progressively greater influence farther from the root. These factors include microbe-microbe interactions, soil type, pH, water content, nutrient availability, geographic location, agricultural practices, and the presence of contaminants (Yeoh et al. 2016; Uribe et al. 2022).

## Root metabolites as selective forces in microbiome assembly

Plants are estimated to produce between 0.1 and 1 million metabolites, more than any other kingdom of life (Fang et al. 2019). This extraordinary chemical diversity poses a significant challenge for mechanistically understanding how metabolites mediate root microbiome assembly. The complexity arises in part from the highly dynamic and context-dependent nature of plant metabolic profiles. Factors such as developmental stage, physiological status, and exposure to abiotic or biotic stresses can all influence metabolite production (Chaparro et al. 2013; Vismans et al. 2022; Pantigoso et al. 2025). Root architecture further contributes by affecting both the synthesis and spatial patterns of metabolite exudation (McKay Fletcher et al. 2020). Exudation also varies along the root's longitudinal axis (Loo et al. 2024) and across different root cell types (Froschel et al. 2021). Once released into the rhizosphere, metabolites differ in their stability and diffusion rates, which in turn shape their microbial impact (Kuzyakov and Razavi 2019). Despite these layers of complexity, the plant's genotype ultimately constrains its metabolic capacity. Thus, while environmental and developmental factors modulate the metabolome in situ, its overall potential is genetically determined.

The composition of root exudates is highly diverse, encompassing a wide range of chemical classes that influence rhizosphere microbial communities. Exudates consist primarily of high-molecular-weight compounds, mainly polysaccharide mucilage and proteins, and a smaller but chemically diverse fraction of low-molecular-weight metabolites (Li et al. 2024). These low-molecular-weight compounds can be classified based on characteristics such as volatility or solubility (Kuzyakov and Razavi 2019), chemical structure (Domingo-Fernandez et al. 2023), metabolic origin (primary vs secondary; Fernie et al. 2024), or ecological function, such as nutrition, signaling, or defense (Gasperini and Howe 2024).

Broadly, primary and secondary metabolites play distinct yet complementary roles in microbiome assembly. Primary metabolites, including sugars, amino acids, and organic acids, are essential for plant growth and serve as key carbon and nutrient sources for microbes in the rhizosphere. Their exudation patterns influence microbial abundance and activity, often favoring fast-growing, copiotrophic organisms (Lopez et al. 2023). Microbes capable of metabolizing specific plant-derived carbon sources, such as inositol, are often among the most rhizosphere competent (O'Banion et al. 2023; Sánchez-Gil et al. 2023). Moreover, microbial preference for 1 carbon source can result in a beneficial microbe overgrowing deleterious ones and may also trigger microbial chemotaxis and biofilm formation (Rudrappa et al. 2008; Boubsi et al. 2023). For example, the exudation of malic acid by Arabidopsis can specifically recruit the beneficial bacterium *Bacillus subtilis* FB17 (Rudrappa et al. 2008). However, malic acid can also induce the production of effectors in pathogenic microbes (Cao et al. 2025). Consequently, plants must tightly regulate sugars levels in the apoplast to control bacterial growth (Nakagami et al. 2024).

In contrast, secondary metabolites, such as flavonoids, coumarins, glucosinolates, benzoxazinoids, terpenes, saponins, alkaloids, and indole-derived compounds, are not directly involved in core metabolism but share overlapping functions in nutrient mobilization, microbial recruitment, signaling, and defense (Pascale et al. 2020; Jacoby et al. 2021; Rizaludin et al. 2021; Korenblum et al. 2022; Zhou et al. 2024). Due to their specificity, secondary metabolites contribute to genotype-dependent shaping of the root microbiome. The interplay between primary and secondary metabolite exudation forms the basis of a metabolite-mediated selection process that helps plants assemble beneficial microbial communities while defending against potential pathogens (Bisht et al. 2025).

Other plant traits, such as root morphology, gas exchange, and the release of root cap border cells, also impact microbial composition (Reinhold-Hurek et al. 2015), though their influence is closely linked to metabolite production and distribution. For example, root architecture affects both the quantity and spatial pattern of metabolite exudation (Akatsuki and Makita 2020). Moreover, root hair mutants of barley have been shown to host a less diverse microbial community (Robertson-Albertyn et al. 2017), and Arabidopsis mutants lacking endodermal barriers exhibit altered colonization by the beneficial rhizobacterium *Pseudomonas simiae* WCS417 (Verbon et al. 2023). Root border cells, which show elevated secondary metabolic activity, are released in increased numbers during pathogen attack, likely shifting the abundance and composition of exuded metabolites (Sasse et al. 2018; Kranawetter and Sumner 2025). These examples highlight the central role of plant metabolites in structuring rhizosphere microbial communities and emphasize the dynamic feedback mechanisms governing plant-microbe interactions in the root environment.

## The plant immune system: gatekeeper of microbial entry

**Figure 1.**
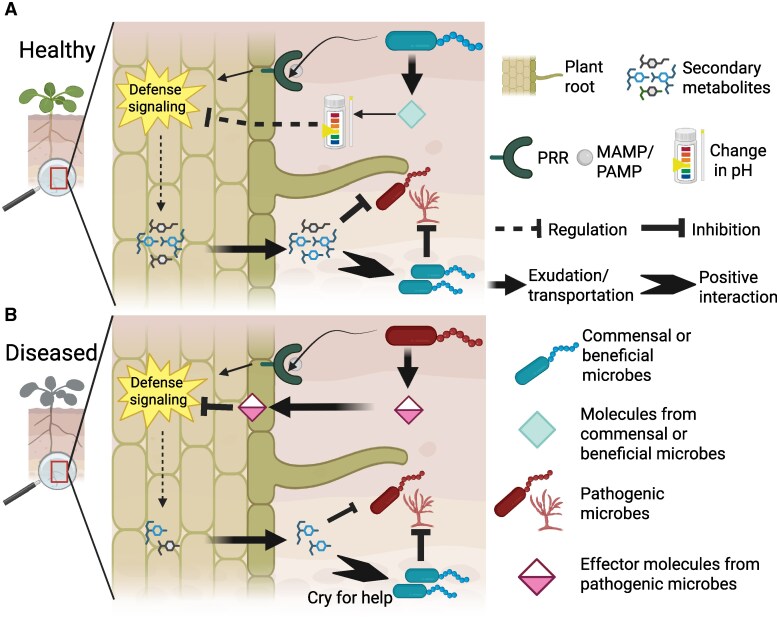
Feedback mechanisms in the rhizosphere between microbes and the plant immune system. Plant defense signaling pathways are activated upon recognition of MAMP or PAMP by surface receptors on plant cells. **A)** Activation of these defense signaling pathways strengthens structural barriers and stimulates the production of antimicrobial secondary metabolites, thereby inhibiting pathogen proliferation. In healthy plants, commensal or beneficial root-associated microbes can modulate root immune responses—such as by lowering environmental pH—to promote their own colonization while preventing excessive immune activation and minimizing defense-growth trade-offs. **B)** Pathogenic microbes can interfere with plant defense signaling by releasing effector molecules that suppress host immunity and promote infection. This interference may alter the profile of defense-related metabolites synthesized and exuded by the plant, often leading to reduced production of antimicrobial compounds. However, these changes may also serve as a signal to the root microbiome, indicating that the plant is under attack and triggering a “cry for help” response that facilitates the recruitment of protective microbial communities. Created in BioRender https://BioRender.com/oaixjpd.

The mechanisms by which plants recognize harmful from beneficial microbes in the soil ecosystem is one of the key topics in plant-microbe interactions research (Sasse et al. 2018; Thoms et al. 2021; Zhang and Kong 2022). The biological system responsible for microbial perception, signal transduction, and response is the plant immune system. From seed emergence to post-harvest, the plant immune system is continuously challenged by microbes. When plant cells initially come into contact with microbes, transmembrane pattern recognition receptors recognize widely conserved microbial epitopes known as microbe- or pathogen-associated molecular patterns (MAMPs/PAMPs; Fig. 1) (Jones et al. 2024). Upon recognition of a microbial MAMP, a broad spectrum of plant defense mechanisms is activated, such as the synthesis of defense-related hormones and antimicrobial secondary metabolites (phytoalexins), as well as the reinforcement of structural barriers through lignification and callose deposition (Millet et al. 2010). The coordinated activation of this first line of defense is collectively referred to as pattern-triggered immunity (PTI). PTI plays a crucial role in deterring generalist pathogens, regulating commensal microbial populations, and preventing dysbiosis (Nakagami et al. 2024).

Since the rhizosphere is a microbial hotspot and therefore rich in MAMPs, tight regulation of root immune responses is essential to prevent constitutive activation of immunity and the resulting growth-defense tradeoffs. Importantly, microbes are not passive entities in this process. Microbe-microbe interactions, as well as the specific pool of microbial functions originally present in the bulk soil, play crucial roles in determining whether a plant will remain healthy or develop disease (Carrion et al. 2019; Wei et al. 2019; Spooren et al. 2024). Several studies have shown that up to 40% of root-associated microbes can locally suppress host immune responses (Fig. 1A) (Yu et al. 2019; Ma et al. 2021; Teixeira et al. 2021; Ordon et al. 2025)—for example, by secreting metabolites such as gluconic acid, which lowers the local environmental pH (Yu et al. 2019). This acidification impairs immune recognition, thereby facilitating microbial colonization and supporting normal plant growth in the MAMP-rich environment. This highlights suppression of local root immunity as a crucial function of the root microbiome.

A second layer of plant immunity enables the recognition of specialized microbial virulence factors, known as “effectors.” These effectors are often used by pathogens to suppress plant defenses or manipulate defense hormone signaling. However, when effectors are detected by plant nucleotide-binding leucine rich repeat receptors, a defense response known as effector triggered immunity is activated (Lonjon et al. 2024). Interestingly, several beneficial microbes also secrete effector-like molecules that modulate host immunity to enable colonization without triggering full immune activation. Unlike effectors from pathogens, which typically suppress host defenses to promote disease (Landry et al. 2020), effectors from symbiotic microbes facilitate mutualistic interactions by fine-tuning host responses (Zamioudis and Pieterse 2012).

Plant hormone signaling pathways are key regulators of defense responses activated during both PTI and effector triggered immunity. Among these, defense signaling metabolites salicylic acid (SA) and jasmonic acid (JA) serve as central regulators, mediators (often antagonistic) of plant immune responses (Pieterse et al. 2014; Aerts et al. 2021). SA biosynthesis and signaling are typically induced by biotrophic pathogens and sap-feeding insects, whereas the JA response is predominantly activated by necrotrophs and insect herbivores (Aerts et al. 2021). This differential activation has important implications for pathogen success: biotrophs benefit from suppression of cell death and senescence, while necrotrophs may exploit pathways that promote these processes (Kazan and Lyons 2014). A well-studied case is *Pseudomonas syringae* pv. *tomato* DC3000, which produces the effector coronatine, a structural mimic of JA, with SA-suppression activity that facilitates infection (Xin and He 2013).

The defense-related hormones SA and JA have also been shown to influence microbiome assembly (Carvalhais et al. 2015; Lebeis et al. 2015) by regulating the biosynthesis of secondary metabolites, such as glucosinolates (Kudjordjie et al. 2021), as well as through their own selective antimicrobial activity (Arenas-Castro et al. 2016; van der Meij et al. 2023). These hormone pathways differentially modulate the production of defense-related metabolites. For instance, glucosinolates, a class of sulfur-containing phytoalexins with antimicrobial properties, vary in composition depending on whether SA or JA signaling predominates (Mikkelsen et al. 2003). Such variation in antimicrobial profiles can influence microbial sensitivity and drive predictable shifts in microbiome composition (Fig. 1B) (Unger et al. 2024). Changes in root exudation profiles of pathogen-infected plants have been shown to recruit specific protective microbiota to the root system, which can, in turn, induce systemic resistance against the invading pathogen (Berendsen et al. 2018; Yuan et al. 2018; Rizaludin et al. 2021; Vismans et al. 2022; Goossens et al. 2023). Thus, infected plants may issue a “cry for help” through the secretion of secondary metabolites that play a role in shaping and mobilizing their root-associated microbiome (Fig. 1B).

Interestingly, plant hormones can also directly affect microbial physiology. In *Streptomyces*, low concentrations of JA were found to enhance the production of the polyketide antibiotic actinorhodin, whereas higher concentrations were toxic (van der Meij et al. 2023). Additionally, SA was shown to be necessary for the growth of some Streptomyces and Actinobacteria strains on minimal medium (Lebeis et al. 2015). These examples illustrate that plant hormones can have a significant impact on members of the plant microbiome.

Besides the above-mentioned defense hormones and glucosinolates, several other classes of secondary metabolites involved in defense play key roles in shaping root-associated microbial communities by modulating both microbial recruitment and defense. Camalexin, the primary phytoalexin in Arabidopsis, is an indole-derived antimicrobial compound produced in response to pathogen challenge. While primarily studied for its role in defense, recent findings suggest that camalexin can influence rhizosphere microbial activity and nurture beneficial microbes (Koprivova et al. 2019). Similarly, terpenes, steroidal saponins, and alkaloids comprise compounds that can function as antimicrobials or chemoattractants, thereby influencing microbial survival and colonization (Huang et al. 2019; Nakayasu et al. 2021; Takamatsu et al. 2023; Zhou et al. 2024).

Specific groups of metabolites are often unique to or significantly enriched in certain plant families, and these compounds can strongly influence whether a plant can host or associate with a particular microbe (Wang et al. 2019). Examples of metabolite classes that are predominantly distributed in particular families include glucosinolates in the Brassicaceae, steroidal glycoalkaloids in the Solanaceae, and isoflavones in leguminous species (Wang et al. 2019). These lineage-specific compounds not only serve defensive roles but also contribute to shaping the rhizosphere microbiome through a balance of antimicrobial defense and selective microbial recruitment, tailored to both environmental conditions and plant genotype. To overcome this chemical barrier of antimicrobial root metabolites, specialized rhizosphere microbes have evolved the ability to metabolize or degrade these compounds. Gene clusters encoding the necessary enzymes have been identified—for example, in a *Sphingobium* species that metabolizes specific benzoxaxinoids (Thoenen et al. 2024), in another *Sphingobium* species that degrades saponins (Nakayasu et al. 2023), and in a *Variovorax* species that degrades isoflavones (Aoki et al. 2024).

## Root metabolites as dual regulators of nutrient acquisition and microbiome assembly

**Figure 2.**
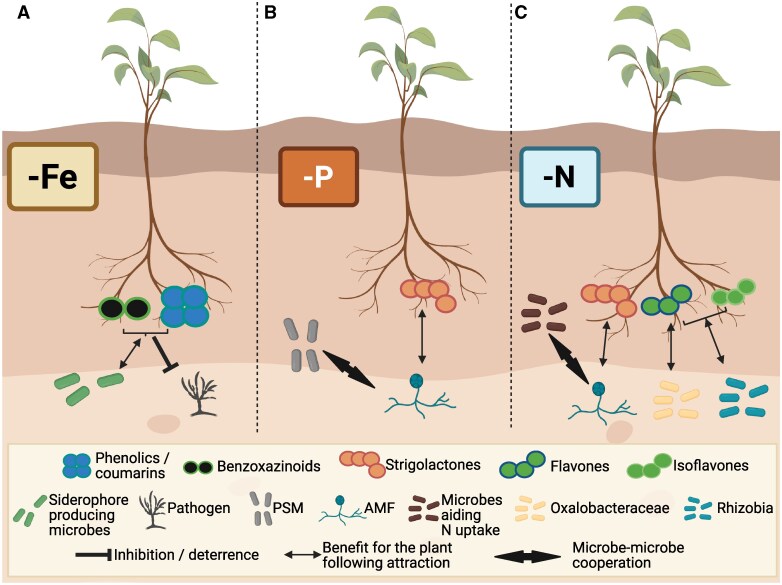
Key root metabolites triggered by nutrient deficiency with roles in the recruitment and association with beneficial microbes. **A)** Under Fe deficiency, plants release a blend of exudates including benzoxazinoids in grasses and phenolics in other plant species. Among the phenolics, coumarins have selective antimicrobial properties, shaping the root microbiome—for example, by inhibiting specific pathogenic microbes while minimally affecting growth of various beneficial rhizobacteria that support Fe mobilization, such as microbes with siderophore production traits. **B)** During P limitation, plants secrete strigolactones that can specifically attract AMF, which assist in P acquisition and form associations with phosphate-solubilizing microbes (PSM). **C)** In N-limited conditions, plants also exude strigolactones and, additionally, flavones and isoflavones. Flavones attract bacteria from the family Oxalobacteraceae, which support N uptake, and both flavones and isoflavones mediate symbiosis with Rhizobia depending on the legume species, leading to atmospheric N fixation for the plant. Mycorrhiza can also cooperate with microbes that can aid nitrogen uptake. Created in BioRender: https://BioRender.com/jjhiqh0.

Many plant-derived metabolites play a dual role in supporting plant health by both enhancing nutrient acquisition and shaping the composition of root-associated microbial communities. In response to nutrient limitations, plants secrete specific metabolites that not only mobilize scarce nutrients but also act as selective agents for microbial recruitment (Fig. 2). For example, under iron (Fe) deficiency, plant roots exude phenylpropanoid-derived coumarins such as fraxetin, redox-active metabolites that reduce insoluble ferric Fe (Fe^3+^) into its more bioavailable ferrous form (Fe^2+^) (Stringlis et al. 2019). Beyond their role in Fe mobilization, coumarins exhibit selective antimicrobial activity, contributing to the assembly of beneficial rhizosphere microbiomes (Stringlis et al. 2018; Vismans et al. 2022). Coumarin secretion also promotes the enrichment of microbial taxa capable of improving Fe availability through the production of Fe-chelating siderophores (Harbort et al. 2020) and microbial redox-active metabolites such as phenazines, which can also solubilize Fe (McRose et al. 2023). Interestingly, these compounds are adapted to different rhizosphere conditions: microbial phenazines are more active in acidic, low-oxygen niches, whereas plant-derived coumarins retain redox activity under oxic, mildly alkaline conditions. Root exudates such as glucose may further influence the redox potential and functional niche of these metabolites (McRose et al. 2023).

A similar dual function has been described for benzoxazinoids, a class of indole-derived metabolites primarily produced by grasses such as maize and wheat. In the rhizosphere, benzoxazinoids contribute to Fe mobilization by forming soluble Fe^3+^-benzoxazinoid complexes, which facilitate Fe uptake under limiting conditions (Hu et al. 2018a). In parallel, they exert selective antimicrobial activity, shaping root microbiome composition and promoting the enrichment of beneficial bacterial taxa involved in nutrient cycling and pathogen suppression (Hu et al. 2018b; Cotton et al. 2019; Gfeller et al. 2024). Through these combined effects, benzoxazinoids act similarly to coumarins as key chemical mediators that align Fe foraging with the recruitment of a supportive microbiome.

Under phosphate (P) deficiency, plants exude specific metabolites, such as carotenoid-derived strigolactones, which stimulate the recruitment and colonization of beneficial microbes involved in P acquisition. Strigolactones promote the growth and metabolic activation of arbuscular mycorrhizal fungi (AMF), whose hyphal networks extend the functional root surface and facilitate P uptake (Gutjahr and Parniske 2013; Waters et al. 2017). Although AMF themselves have limited capacity to solubilize organic phosphate, they often form synergistic partnerships with P-solubilizing bacteria that colonize their hyphae and secrete phosphatases and organic acids to mobilize unavailable P sources (Nacoon et al. 2020). Also AMF-hyphae-colonizing bacteria that enhance nitrogen (N) uptake have been shown to support AMF-mediated promotion of plant growth (Zhang et al. 2024). In non-mycorrhizal species such as *Arabidopsis*, endophytic fungi like *Colletotrichum tofieldiae* and *Serendipita indica* contribute to P acquisition by inducing host phosphate transporters or improving P-use efficiency (Hiruma et al. 2016).

Under N deficiency, plants similarly adjust root exudation to promote associations with beneficial microbes that support N acquisition. In legumes, low N conditions trigger enhanced exudation of flavonoids such as flavones and isoflavones, which induce the expression of nodulation (*nod*) genes in symbiotic rhizobia, leading to the formation of nitrogen-fixing root nodules (Poole et al. 2018; Fujimatsu et al. 2024). In non-legumes such as maize, N stress leads to the exudation of specific flavones that enrich for Oxalobacteraceae, a bacterial family associated with improved N uptake and lateral root development (Yu et al. 2021). Additional microbial partners include free-living diazotrophs like *Azospirillum*, *Azotobacter*, and *Xanthobacter*, which enhance plant growth and N-use efficiency (Liu et al. 2017; Banik et al. 2019; Zeffa et al. 2019). Other beneficial microbes, including AMF and beneficial endophytes like *S. indica* and *Trichoderma* spp., also support N nutrition by stimulating nitrate transporter expression or enhancing root function (Vahabi et al. 2015; Silletti et al. 2021).

Overall, these coordinated responses illustrate how root exudates help align microbial recruitment with nutrient needs, highlighting the dual role of exudates in nutrient acquisition and microbiome assembly.

## Concluding remarks

As global agriculture faces the dual challenge of improving crop productivity and reducing reliance on unsustainable chemical inputs, harnessing the potential of plant traits and microbiota offers a promising path forward. This review highlights how plant–metabolite–microbiome interactions have coevolved with essential life-support systems such as immunity and nutrient acquisition. Plants use defense-related metabolites to recruit specific beneficial microbiota that help mitigate biotic stress, and they dynamically respond to nutrient limitations by exuding metabolites not only to mobilize scarce nutrients but also to attract beneficial microbial partners that alleviate nutrient stress.

Looking ahead, a deeper understanding of plant–microbiome interactions during biotic stress and abiotic stresses such as nutrient limitation will be essential for developing sustainable, microbiome-informed strategies to enhance plant health and nutrition (see Outstanding questions box). These strategies include plant-driven approaches, such as breeding for traits that enhance specific root exudations and beneficial microbe recruitment, as well as microbe-driven solutions, including the development of designed microbial consortia or engineered microbial strains with improved functional capacities.

Future efforts should prioritize the translation of insights from model species to crops, the integration of multi-omics data with artificial intelligence and machine learning tools, and the validation of innovations under realistic field conditions. Past parallel metabolome-microbiome experiments have been instrumental in uncovering beneficial plant–metabolite–microbiome interactions (Stringlis et al. 2018; Zhalnina et al. 2018; Jacoby et al. 2021; Pang et al. 2021; Csorba et al. 2022; Hong et al. 2022). Next-generation spatial omics technologies are now pushing resolution from whole-root averages to micrometer-scale niches. For instance, spatial metatranscriptomics applied to outdoor-grown Arabidopsis leaves revealed bacterial and fungal “hotspots” and linked them to localized induction of plant defense genes. Spatial mass spectrometry imaging platforms have also been developed to map the distribution of root exudates and microbial metabolites directly in the rhizosphere, revealing fine-scale chemical gradients and plant–microbe interfaces (Saarenpää et al. 2024; Veličković et al. 2024).

Deep genome and metagenome sequencing underpin constraint-based community metabolic models. Successfully applied in human microbiome studies, for example, to predict microbial drug transformations in personalized medicine, these models are now being adopted in plant microbiome research. They are helping to elucidate how carbon partitioning and cross-feeding shape microbiome assembly, with broad potential applications (Heinken et al. 2023; Schäfer et al. 2023; Blonde et al. 2025). Complementing these flux-based models, Li et al. (2025) introduced RhizoSMASH, a genome-synteny algorithm that mines bacterial genomes for catabolic gene clusters, enabling the prediction of which strains can catabolize specific root exudates and thrive in the rhizosphere.

To move from correlation to causation, future studies should generate co-registered multi-omics datasets from the exact same samples and integrate them with causal-inference ML frameworks capable of disentangling directional plant–microbe feedbacks (Xu et al. 2021). The rapid development of AI and ML offers significant opportunities, as plant microbiome-based predictive models are beginning to show strong potential for forecasting plant performance from large microbiome datasets (Wei et al. 2019; Kang et al. 2022; Lutz et al. 2023; Song et al. 2025). By integrating these technological advances with plant genetics, microbial ecology, and biotechnology, we can design resilient agroecosystems that reduce reliance on fertilizers while improving crop nutrition and health.

## Data Availability

Not applicable.

## Dive Curated Terms

The following phenotypic, genotypic, and functional terms are of significance to the work described in this paper:

root AmiGo: PO:0009005

camalexin CHEBI: CHEBI:22990

coumarin CHEBI: CHEBI:28794

plant hormone CHEBI: CHEBI:37848

flavones CHEBI: CHEBI:24043
